# Assessment of Myocardial Contractile Function Using Global and Segmental Circumferential Strain following Intracoronary Stem Cell Infusion after Myocardial Infarction: MRI Feature Tracking Feasibility Study

**DOI:** 10.5402/2013/371028

**Published:** 2012-09-29

**Authors:** Sabha Bhatti, Hussein Al-Khalidi, Kan Hor, Abdul Hakeem, Michael Taylor, Arshed A. Quyyumi, John Oshinski, Andrew L. Pecora, Dean Kereiakes, Eugene Chung, Gianni Pedrizzetti, Tomasz Miszalski-Jamka, Wojciech Mazur

**Affiliations:** ^1^University of Cincinnati Hospital, Cincinnati, OH, USA; ^2^Duke University School of Medicine, Durham, NC, USA; ^3^Cincinnati Children's Hospital Medical Center, Cincinnati, OH, USA; ^4^Emory University, Atlanta, GA, USA; ^5^Amorcyte Inc., Hackensack, NJ, USA; ^6^The Christ Hospital Heart and Vascular Center, Suite 138, 2123 Auburn Avenue, Cincinnati, OH 45219, USA; ^7^University of Trieste, Trieste, Italy; ^8^Center for Diagnosis Prevention and Telemedicine, John Paul II Hospital, Krakow, Poland

## Abstract

*Background*. Magnetic resonance imaging (MRI) strain analysis is a sensitive method to assess myocardial function. Our objective was to define the feasibility of MRI circumferential strain (*ε*_cc_) analysis in assessing subtle changes in myocardial function following stem cell therapy. *Methods and Results*. Patients in the Amorcyte Phase I trial were randomly assigned to treatment with either autologous bone-marrow-derived stem cells infused into the infarct-related artery 5 to 11 days following primary PCI or control. MRI studies were obtained at baseline, 3, and 6 months. *ε*_cc_ was measured in the short axis views at the base, mid and apical slices of the left ventricle (LV) for each patient (13 treatments and 10 controls). Mid-anterior LV *ε*_cc_ improved between baseline −18.5 ± 8.6 and 3 months −22.6 ± 7.0, *P* = 0.03. There were no significant changes in *ε*_cc_ at 3 months and 6 months compared to baseline for other segments. There was excellent intraobserver and interobserver agreement for basal and mid circumferential strain. *Conclusion*. MRI segmental strain analysis is feasible in assessment of regional myocardial function following cell therapy with excellent intra- and inter-observer variability's. Using this method, a modest interval change in segmental *ε*_cc_ was detected in treatment group.

## 1. Introduction

Circumferential strain (*ε*_cc_) analysis is an established method to assess myocardial function. *ε*_cc_ has been demonstrated to detect changes in myocardial contractility across a variety of cardiac conditions including hypertensive [1] or hypertrophic cardiomyopathy [2] and Duchenne muscular dystrophy [3] before changes in left ventricular ejection fraction (LVEF) are observed. Cell therapy offers a promising approach for regeneration of damaged vascular and cardiac tissue after acute myocardial infarction (MI) [4–8]. The Amorcyte trial evaluated effect of autologous bone marrow derived CD34+ cell therapy on LVEF and myocardial perfusion [9]. Significant improvement in myocardial perfusion and a trend towards improvement in LV ejection fraction were reported. Because the changes seen in systolic function are modest and by design regional, the more sensitive myocardial strain techniques offer an attractive option for analyzing these results. Accordingly, we evaluated the feasibility of MRI-derived segmental *ε*_cc_ analysis in patients treated with cell therapy following primary intervention for ST-segment elevation myocardial infarction (STEMI), using the feature tracking (FT) technique. The FT technique was previously validated for *ε*_cc_ assessment against harmonic phase imaging (HARP) [10] and subsequently utilized for assessment of cardiac involvement in Churg Strauss disease [11] and in dobutamine cardiac MRI stress testing [12]. One of the major advantages of this technique is that additional imaging is not needed, as analysis is performed on cine (steady-state free precession, SSFP) images and does not require tagging.

## 2. Methods

### 2.1. Study Population and Protocol in the Amorcyte Study

Subjects with STEMI who were treated with intracoronary stent implantation within 3 days of infarction and an LVEF ≤50% by echocardiography demonstrating regional wall motion abnormality in the distribution of the infarct-related artery ≥4 days after stenting were enrolled [9]. Briefly, the treatment groups patients were treated with escalating dose of stem cells (5, 10, and then 15 million CD34+ cells) and compared with control. At 24–48 hours after bone marrow harvest, CD34+ cells were infused into the infarct-related artery through an over-the-wire balloon catheter placed within the previously stented segment using a stop flow technique [13].

### 2.2. MRI Imaging

All studies were performed using 1.5 Tesla MRI scanners. Cardiac functional imaging was performed with retrospective electrocardiogram (ECG) gating, utilizing a standard SSFP technique, LV end-systolic and end-diastolic volumes, LVEF, and infarct size at baseline, 3 months, and 6 months were assessed as described previously [9].

### 2.3. Image Analysis

Using Diogenes CMR FT software (TomTec Imaging Systems, Munich, Germany), global *ε*_cc_ was measured in left ventricular short axis steady-state free precession (SSFP) views at base, mid, and apex for each patient at baseline, 3 months, and 6 months by 2 independent readers who were blinded to treatment assignment. In addition, segmental *ε*_cc_ was measured for all individual mid-ventricular segments (segments 13 through 18), Segmental strain analysis was not undertaken in apical and basal segments due to low reproducibility (for apical segments) and no infarcted tissue present (basal segments). Thirteen treatments and 10 controls underwent strain analysis. 

### 2.4. Diogenes CMR FT Software

Diogenes CMR FT software (TomTec Imaging Systems, Munich, Germany) is a vector-based analysis tool based on a hierarchical algorithm that operates at multiple levels using a combination of 1-dimensional and 2-dimensional tracking techniques. Based on a contour manually drawn by an expert reader along the LV endocardial border frame, the software automatically propagated the contour and followed its features throughout the remainder of the cardiac cycle. Features tracked in each voxel (the 3-dimensional analogue of a pixel) by the software include brightness gradient at the tissue-cavity interface in homogeneities of the tissue (with respect to a 256-level gray scale), geometrical “roughness” of the tissue edges, and additional specific anatomical elements (papillary muscles and septum). Tracking is based on optical flow algorithms applied on sequentially reduced windows to ensure resolving large amplitude displacements and still discern local differences, and also implicitly accounts for spatial coherence and time-periodic motion. The tracking over all frames of a set of individual material points along the border permits to quantify their frame by frame displacement. The circumferential strain is defined as the instantaneous local border lengthening or shortening: if *L*(*t*) indicates the distance between two neighboring points along the border, the strain is computed as *St*(*t*) = (*L*(*t*) − *L*_*R*_)/*L*_*R*_, where *L*_*R*_ indicates such length at the ECG R wave. The systolic strain is therefore (*L*_*ES*_ − *L*_*R*_)/*L*_*R*_. This represents the Lagrangian strain and it is the most frequently used in cardiac imaging [14–16]. Strain values are computed at 48 points along the border, the entire border is then automatically subdivided into six segments of same length and segmental strain is computed as the average strain of all the points falling in that segment. The global strain is then defined as the average of all segmental strain values. Analysis can be accomplished very quickly, similar to speckle tracking in echocardiography [17] (this reference includes video of actual FT procedure).

### 2.5. Statistical Analysis

Global circumferential strain data were summarized as mean ± SD as well as the median. Due to small sample size, comparisons within each group in change from baseline for the global circumferential strain at 3 months and 6 months were conducted using the Wilcoxon signed-rank test. Similarly, change from baseline in circumferential strain at mid-anterior wall at 3 months and 6 months was conducted using the Wilcoxon signed-rank test. 

Bland and Altman [18] analysis was used to test the interobserver and intraobserver agreement for global circumferential strain at base, mid, and apex. The test statistic [19] for testing the readings agreement between observer 1 and observer 2 was based on $t=r\left(\sqrt{k-2}/\sqrt{1-r^{2}}\right)$, where *t* follows the *t* distribution with *k*-2 degrees of freedom, *r* is the correlation between the average readings ([observer 1 + observer 2]/2) and the difference (observer 1 – observer 2) with zero or small *r* indicating an agreement between methods (i.e., no systematic bias between readers), and *k* is the paired sample size.

Infarct size as a function of dose and changes in mid anterior strain at baseline, month 3, and month 6 was analyzed using analysis of variance (ANOVA). 

Statistical significance was set at *P* < 0.05. Statistical analysis was performed using SAS software version 9.2 (SAS Institute, Inc., Cary, NC). 

## 3. Results

Most patients (9/10 in the control group and 12/13 in the treatment group) had evidence of anterior wall myocardial infarction by late gadolinium enhancement (LGE). There was one lateral infarct in the control group and one inferior in the treatment group.

**Figure 1:**
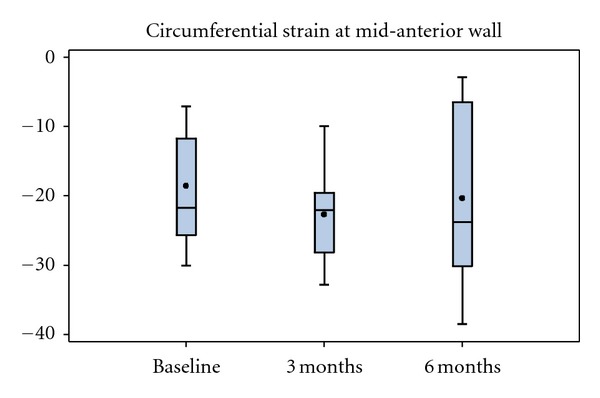
Segmental strain of mid-anterior (MA) segments at baseline, 3, and 6 months followup in the treatment group. Improvement noted at 3 months (*P* = 0.03) was no longer present at 6 months (*P* = 0.34).

No difference was detected in the treatment or control groups in global *ε*_cc_ at any level, except for the basal slice in the treatment group, the change from baseline and 3 months was statistically significant (−18.4 ± 14.4 versus −24.4 ± 4.8, *P* = 0.03, Table 1). Mid-anterior *ε*_cc_ in the treatment group showed improvement between baseline −18.5 ± 8.6 and 3 months −22.6 ± 7.0, *P* = 0.03 with no further improvement at 6 months −20.3 ± 12.4, *P* = 0.34 (Figure 1). There was no statistically significant difference in mid-anterior segments of the control group (−19.7 ± 7.3 to −18.6 ± 10.7, *P* = 0.91) at 3 months and no changes were observed at 6 months (−19.7 ± 7.3 to −23.0 ± 8.4, *P* = 0.36). No changes were noted in any other segments in the mid-ventricular short axis between any time points.

Bland and Altman analyses showed an excellent interobserver agreement for global *ε*_cc_ at basal, mid, and mid segmental with (Pearson's correlation between average and difference readings *r*) *r* = −0.03, *P* = 0.83, *r* = 0.10, *P* = 0.44, and *r* = 0.08, *P* = 0.14, respectively. However, there was a poor interobserver agreement on apex with *r* = −0.40, *P* = 0.0009. In addition, there was an excellent intraobserver agreement for global *ε*_cc_ at basal, mid, and apex with *r* = −0.07, *P* = 0.69, *r* = 0.19, *P* = 0.29, and *r* = 0.26, *P* = 0.14, respectively.

In the treatment group, ANOVA analysis showed no significant difference between the doses of stem cell, size of infarct at baseline, and changes in mid-anterior *ε*_cc_ at baseline, Month 3, and Month 6. As previously reported, there were no significant differences in the change in infarct size or LVEF in treatment versus control groups after 3 or 6 months.

**Figure 2:**
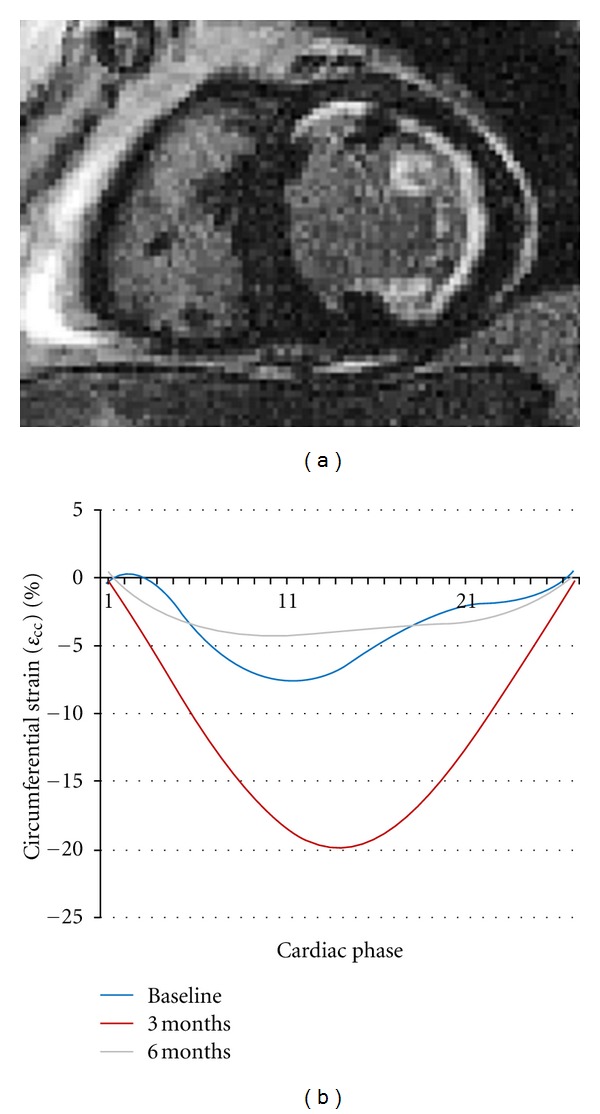
Late gadolinium enhancement and mid-anterior *ε*_cc_ for baseline, 3, and 6 months. There was initial improvement at 3 months followup, but deterioration at 6 months, due to left ventricular remodeling.

Example of patient in the treatment group is presented in Figure 2.

## 4. Discussion

The major finding of this substudy from the Amorcyte-randomized trial was feasibility of detection of segmental *ε*_cc_ changes using FT technique in patients treated with intracoronary bone-marrow-derived CD34+ cell infusion after acute ST elevation MI. This is of importance as FT analysis does not require additional imaging (such as tagging) and it can be used retrospectively as cine sequences are obtained in all studies while tagging is typically not a part of the standard protocol. FT technique is specifically designed for MRI analysis and differs from recently reported velocity vector imaging (VVI) software (which was designed for echocardiographic speckle tracking) [20]. In addition, we demonstrated that patients who received cell therapy had a statistically significant improvement in the mid-anterior *ε*_cc_ without significant changes in LVEF at 3 but not at 6 months. As most of the analyzed cohort suffered anterior wall MI, the improvement in *ε*_cc_ within this infarcted region was likely driven by the beneficial effects of cell therapy. Finally, both inter- and intraobserver agreement, for strain analysis were excellent for mid and basal segments. Interestingly, intraobserver (but not interobserver) variability was poor for apical segment; possible explanation is presence of apical trabeculations, which have been defined as endocardium by one reader versus compact myocardium used by other reader.

Improvement in 2D echo-derived LV strain has been reported by Herbots et al. [21] who demonstrated significant improvement in the global longitudinal strain rate in patients undergoing cell therapy following acute MI. Strain rate improved in infarcted areas and appeared to be a more sensitive marker of response to therapy than was change in global left ventricular EF. Similarly, Plewka et al. [22] reported improvement in systolic strain measurements involving infarcted segments in patients who received cell therapy. Karatasakis et al. [23] reported improvement in longitudinal strain in patients with prior anterior wall MI following cell therapy in the absence of concomitant revascularization. The current study is the first, to our knowledge, to demonstrate the utility of MRI FT-derived segmental *ε*_cc_ in the settings of stem cell therapy. Furthermore, the broad spectrum of data available from MRI (valve assessment, LVEF, infarct mass by LGE, and strain) may obviate the need for echocardiography following cell therapy after MI. Finally a recent study by Hopp et al. [24] which is a substudy of Autologous Stem Cell Transplantation in acute myocardial infarction trial (ASTAMI) explored global and regional myocardial function after intracoronary injection of autologous mononuclear bone marrow cells in acute anterior wall myocardial infarction treated with percutaneous coronary intervention using MRI tagging. The authors found that intracoronary injection of autologous mononuclear bone marrow cells did not influence regional or global myocardial function in this substudy.

Segmental *ε*_cc_ may be an attractive and sensitive surrogate endpoint for evaluation of benefits of stem cell therapy. A recent meta-analysis of postinfarct stem cell trials concluded that changes in LVEF were very modest and strain analysis may be amore useful and sensitive endpoint [25]. Indeed, in the present analysis, infarcted (and treated) mid-anterior segmental *ε*_cc_ improved significantly in the absence of an appreciable change in LVEF. Although the clinical significance of improvement in segmental *ε*_cc_ in the absence of a more global increase in LV contractility is yet to be determined; such changes may predict a beneficial response to therapy with respect to LV remodeling and the degree of infarct zone electrical heterogeneity (which may be associated with increased risk for ventricular reentrant arrhythmias) [26]. Further studies examining the relationship of *ε*_cc_ to both global measures of LV volume or function over time and to meaningful clinical outcomes are clearly warranted.

## 5. Limitations

 Small study population also limited the evaluation of a relationship between infarct transmurality, heterogeneity, presence of microvascular obstruction, myocardial hemorrhage, and the subsequent likelihood of response to stem cell therapy. Further improvement in FT software resolution will be needed to assess changes in *ε*_cc_ in the peri-infarct zone (“gray zone”) which is more likely to provide a favorable environment for infused stem cells than the fully infarcted tissue. Additionally, only mid-myocardial strain was analyzed; it is possible that additional changes would be detected with analysis of endocardial strain. As this method relies on “perfect alignment” of analyzed SSFP images at baseline and followup, a small misalignment may miss the edge of infarct and affect the results. In addition, LGE images need to be obtained at exactly the same level as SSFP images, in order to assess changes in contractility in the infarct and peri-infarct regions.

## 6. Conclusions

In conclusion, FT method can detect regional changes in *ε*_cc_ following intracoronary stem cell infusion after myocardial infarction. The relationship between these variable and clinical outcomes requires further study.

## Figures and Tables

**Table 1:** Global circumferential strain at base, mid, and apex for cases and controls.

Strain	Mean	SD	Median	*P* value*
		Treatment		

Base				
Baseline	−18.4	14.4	−22.0	
Month 3	−24.4	4.8	−24.9	0.03
Month 6	−22.8	6.7	−21.8	0.63
Mid				
Baseline	−18.2	12.3	−21.1	
Month 3	−20.9	5.5	−21.4	0.59
Month 6	−20.7	6.9	−22.5	0.30
Apex				
Baseline	−16.2	6.3	−15.4	
Month 3	−16.6	6.4	−15.8	0.79
Month 6	−18.3	7.8	−19.9	0.35

		Control		

Base				
Baseline	−21.3	6.3	−20.0	
Month 3	−21.7	4.9	−21.0	0.73
Month 6	−22.8	4.8	−20.6	0.30
Mid				
Baseline	−20.4	6.2	−19.5	
Month 3	−19.1	6.1	−18.3	0.99
Month 6	−20.8	5.9	−18.9	0.91
Apex				
Baseline	−20.3	9.0	−17.2	
Month 3	−21.1	8.1	−20.9	0.73
Month 6	−23.0	6.2	−23.5	0.25

*Wilcoxon signed-rank test for change from baseline within each group.
